# Supplementary material for: Geographical indications and health-conscious behaviors among nursing students: A mixed methods study
Source: PLoS One. 2026 Mar 5;21(3):e0344242. doi: 10.1371/journal.pone.0344242 (PMC12962537; doi:10.1371/journal.pone.0344242)
Supplement: Attachment — Submitted filename: Geographical indicators nutrition.docx [file pone.0344242.s002.docx]

**The Role of Geographical Indications in Health-Conscious Behaviors: Mixed Method Evidence from Nursing Students**

**Esin KAVURAN^1^**

**^1^**Ataturk University, Turkey

**e-mail:** [esin.kavuran@atauni.edu.tr](mailto:esin.kavuran@atauni.edu.tr)

**Phone: +90537 430 23 19**

**ORCID:** <https://orcid.org/0000-0002-9825-8979>

**Erkan DENK^2^**

**^2^** Bitlis Eren University, Turkey

**e-mail:** [edenk@beu.edu.tr](mailto:edenk@beu.edu.tr)  **(corresponding author)**

**Phone:** +90532 636 36 06

**ORCID:** <https://orcid.org/0000-0002-2144-3316>

**ABSTRACT**

This study examined the relationship between nursing students' awareness of Geographically Indicated (GI) certified products, mindful eating behaviors, and healthy eating attitudes. Using the Theory of Planned Behavior as the framework, it was hypothesized that positive attitudes toward GI products, shaped by social norms and perceived behavioral control, would enhance students' healthy eating behaviors. A cross-sectional study was conducted with 392 nursing students from a faculty population of 1,385. Data were collected using scales that measured healthy eating attitudes, mindful eating, and attitudes toward purchasing GI-certified products. The results indicated that younger students (aged 17-25) and females demonstrated more positive attitudes toward GI products, which in turn positively affected their healthy eating behaviors. Additionally, students with more personal knowledge about GI products and those from health-conscious families exhibited healthier eating behaviors. Structural Equation Modeling (SEM) results revealed that attitudes toward GI products and mindful eating significantly contributed to healthy eating behaviors, with mindful eating serving as a moderator in this relationship. Qualitative interviews highlighted the influence of societal and familial factors on students' attitudes toward GI products. However, barriers such as price and accessibility were identified as challenges in purchasing GI products. The study concluded that integrating GI product awareness into nursing education could enhance students' health-conscious behaviors, improving their personal and professional approaches to healthy eating.

**Keywords:** consumer attitudes, consumer behavior, geographical ındications products, healthy eating, mindful eating, nursing students, theory of planned behavior.

**1. Introduction**

According to the World Health Organization (2022), a healthy diet is achieved by consuming adequate amounts of various nutrients that meet the body’s needs. This includes sufficient intake of nutrients such as fruits, vegetables, whole grains, proteins, and healthy fats while limiting the consumption of sugar, salt, and processed foods (Organization, 2022) . Research emphasizes the positive effects of mindful eating on consumer purchasing behavior, weight management, and the adoption of healthier eating habits (Katterman et al., 2014; Tapper, 2022). Consumers with higher awareness levels tend to prefer fresh, organic, and minimally processed foods, opting for products with clear nutritional value and minimal additives (Koca & Arkan, 2021). Geographical Indication (GI) certified products are becoming an important choice for health-conscious consumers. These products are associated with specific regions, produced using traditional methods, and are perceived as more natural, less processed, and nutritionally superior due to their characteristics (Belletti et al., 2017). Studies show that GI-certified products are considered healthier due to their pure ingredients and traditional production processes (Fernández-Zarza et al., 2021; Török et al., 2020). The certification and labeling of GI products significantly impact consumer behavior, increasing consumer confidence and facilitating informed purchasing decisions (Vandecandelaere et al., 2021) . The use of logos and certifications signals quality and authenticity, shaping consumer preferences and contributing to healthy dietary choices (Calboli, 2015). Therefore, GI-certified products are becoming an increasingly attractive option for consumers seeking natural, high-quality food options. In this context, mindful eating plays a critical role in shaping consumer behavior, especially in the health and wellness sector. Mindful eating enables consumers to consciously perceive and control their food choices, guiding them toward healthier purchasing decisions (Tapper, 2022). Consumers with high levels of awareness are more likely to seek out products that align with their health goals and avoid highly processed foods perceived as unhealthy (Bennett & Latner, 2022). To understand these behaviors, a deeper examination of the dynamics influencing consumer behavior is needed. At this point, it becomes important to refer to the Theory of Planned Behavior (TPB). The Theory of Planned Behavior (TPB) focuses on three key factors that influence the likelihood of individuals engaging in a particular behavior: attitudes, subjective norms, and perceived behavioral control. These factors play a crucial role in shaping consumers' motivation and competence to make healthy choices (Conner, 2020). Nursing students, as future healthcare professionals, represent an important consumer group in terms of health-conscious purchasing behavior and attitudes (Mancin et al., 2023; Vasiloglou et al., 2020).

Within the framework of TPB, nursing students’ attitudes toward geographical indication (GI) products strengthen the belief that these products are valuable for healthy eating. The health knowledge and personal experiences students possess lead them to develop positive attitudes toward GI products. These attitudes emerge as a significant factor guiding purchasing behavior. Nursing education, in particular, enhances students' health-related knowledge, contributing to the reinforcement of healthy eating habits.

Subjective norms are shaped by the healthy eating expectations of nursing students' social environments. Family, friends, and colleagues are important social factors that influence students' decisions to make healthy choices. As nursing students bear the responsibility of being role models for their surroundings as healthcare professionals, the influence of social norms in this process becomes even more significant (Vasiloglou et al., 2020).

Perceived behavioral control encompasses nursing students’ competence and access to resources in making healthy eating choices and selecting GI products. This control increases students' confidence in making healthy decisions. Understanding how their mindful eating influences consumer behavior regarding GI-certified products is critical in promoting healthy behaviors in both personal and professional contexts (Mengi Çelik & Semerci, 2022). In this regard, nursing students' knowledge and attitudes toward GI products play a crucial role in developing healthy eating habits and spreading these habits within the community. Therefore, focusing more on GI products and healthy eating in nursing education could help students become more informed consumers and enhance their health consciousness. Despite the existing research on the effects of mindful eating and GI-certified products, the consumer behavior of nursing students, especially in relation to GI products, has not been sufficiently studied. This study aims to fill this gap by examining how nursing students' awareness of GI-certified products influences their healthy eating attitudes and purchasing behaviors.

**1.1. Conceptual Framework**

The Theory of Planned Behavior (TPB) is a theory developed by Ajzen in 1985 and is one of the most widely used theories for understanding the intentions of individuals to perform certain behaviors and the processes through which these intentions are converted into behavior. TPB posits that an individual's behavior is determined by their intentions, which are influenced by three main factors: attitudes toward the behavior, subjective norms, and perceived behavioral control. These three components shape an individual’s intention to perform a behavior, and this intention is the strongest predictor of the behavior (Ajzen, 1991). Attitudes toward the behavior refer to the individual’s positive or negative evaluation of performing a particular behavior, and positive attitudes increase the likelihood of the behavior being performed. Subjective norms express the expectations of the individual’s social environment (family, friends, community) and the impact of these expectations on the individual’s behavior; an individual is more likely to perform a behavior if they believe that their social environment approves of it. Perceived behavioral control refers to the individual’s perception of their ability to perform a specific behavior; if the individual believes they have the necessary resources and abilities to perform the behavior, the likelihood of performing that behavior is higher (Conner, 2020) .

**1.2. Application of TPB to Consumer Behavior and Geographic Indication Products**

TPB is a commonly used theory to explain consumer behavior and offers a strong framework for understanding attitudes and behaviors toward geographic indication (GI) products. GI products are known for their specific production processes and quality tied to a particular geographical area, carrying important values such as health, sustainability, and contribution to local communities for consumers (Emekci, 2019; Denk, 2024). Consumers' positive attitudes toward GI products are based on factors such as the health benefits of these products, their contribution to the local economy, and environmental sustainability. Positive attitudes toward GI products strengthen consumers’ intention to purchase these products. Positive views of GI products from family, friends, and social groups can increase individuals' tendencies to prefer these products (Muça et al., 2021). Consumers are more likely to exhibit similar behaviors when they observe that people in their surroundings support or purchase these products. Accessibility to GI products, pricing, and the consumer’s level of knowledge about these products are significant factors affecting purchasing decisions. Consumers may struggle to perform the purchasing behavior even with positive attitudes if they encounter barriers to accessing these products (Figure 1).

**Figure 1.** Consumer Behavior Toward Geographically Indicated Products Based on the Theory of Planned Behavior

**1.3. TPB and Studies Related to Geographic Indication Products**

The Theory of Planned Behavior has been used in numerous studies to explain consumer behavior. Particularly, TPB has proven to be a strong predictive model in research regarding the purchasing intentions of local and geographic indication products. Many studies have shown that consumers' intentions to purchase local and organic products are influenced by the three main components of TPB (Jarma Arroyo et al., 2020; Užar & Filipović, 2023). Consumers' attitudes toward these products, the norms they receive from their social environment, and their perception of accessibility shape their purchasing intentions. Due to the widespread nature of geographic indication products in Europe, numerous studies have been conducted on consumer attitudes toward these products (Bartoli et al., 2022). A study conducted in Italy showed that consumers’ preference for GI-labeled products is associated with their perception of these products as traditional and high-quality. Positive attitudes of consumers toward these products have been linked to the health benefits and contributions to the local economy of these products (Užar & Filipović, 2023). The importance of GI products in terms of environmental sustainability has also been associated with the subjective norm and perceived behavioral control components of TPB. Environmentally conscious individuals are more likely to prefer these products under the influence of positive social norms regarding GI products. Additionally, their ability to control access to GI products significantly affects their purchasing decisions.

**1.4. Hypotheses and the Role of TPB**

The fundamental components of TPB provide a strong framework for understanding nursing students' attitudes and behaviors toward GI products. The following hypotheses support the applications of TPB in this study:

**H_1_:** Attitudes toward GI products significantly affect nursing students’ healthy eating attitudes.

**H_2_:** Conscious eating practices significantly affect nursing students’ healthy eating attitudes.
Conscious eating is related to the perceived behavioral control component of TPB.

**H_3_:** Attitudes toward GI products and conscious eating practices together significantly affect nursing students’ healthy eating attitudes.

**H_4_:** The attitude toward GI products is moderated by conscious eating practices, impacting nursing students’ healthy eating attitudes.

The components of attitudes toward behavior and perceived behavioral control from TPB support this hypothesis. Conscious eating practices reinforce positive attitudes toward GI products and increase their impact on healthy eating. In this study, the relationships between attitudes towards purchasing Geographically Indicated (GI) products, mindful eating, and healthy eating attitudes were examined using the Structural Equation Model (SEM). Baron and Kenny's (1986) four-step mediation analysis model was used to test the hypotheses of the study (Baron & Kenny, 1986) (Figure 2).

**Figure 2.** Model for Determining the Mediating Role in the Research

In the first step, the aim was to determine whether there is a significant direct relationship between GI product purchasing attitudes and healthy eating attitudes (H_1_) and between healthy eating attitudes (H_2_). In this step, the direct effect of the independent variable (attitude towards purchasing GI products) on the dependent variable (healthy eating attitudes) was examined. In the second step, the effect of the independent variable (GI product purchasing attitude) on the mediating variable and the effect of the mediating variable on the dependent variable were tested (H_3_). At this stage, the effect of GI product purchasing on mindful eating (path a) and the effect of mindful eating on healthy eating attitudes (path b) were analyzed. In the third step, after including the mediating variable (mindful eating) in the model, it was tested whether the effect of the independent variable (GI product purchasing attitude) on the dependent variable (healthy eating attitudes) decreased. In the fourth step, it was tested whether the mindful eating variable played a moderating role in the relationship between GI product purchasing attitude and healthy eating attitudes (H_4_).

**2. METHODS**

**2.1.1. Research Design**

This study was designed as a cross-sectional research to examine the effect of nursing students' attitudes toward purchasing Geographical Indication (GI) certified products and mindful eating on their healthy eating attitudes.

**1. Quantitative component**

**2.1.2. Population and Sample**

The study population consisted of 1385 students enrolled in the Nursing Faculty during the 2023-2024 academic year. The population was determined based on the faculty’s registered student numbers and the academic year. For sample selection, random sampling was useu d, and the sample size was calculated using a formula (n=N.Z².p.(1-p)/(N-1).E²+Z².p.(1-p)) commonly used when the population is known. With a 95% confidence level and a 5% margin of error, the sample size was determined to be approximately 301 participants. During the sampling process, a list of students enrolled in the 2023-2024 academic year was obtained from the faculty administration. From this list, a total of 400 studentso, 100 from each class year, were randomly selected and invited to participate in the study. The study was completed with 392 students, as 8 students did not fully complete the survey.

**2.1.3. Data Collection Instruments**

In this study, a socio-demographic data form prepared by the researchers was used. This form includes demographic information such as students' age, gender, marital status, and place of residence; educational and professional information like class level; family and socio-economic status such as family income level, whether there is a healthcare worker in the family, and family lifestyle and eating habits; personal health and eating habits including self-evaluation of eating habits, body mass index, and physical activity level; and behaviors related to purchasing geographical indication products, including the frequency of choosing these products, knowledge about these products, and reasons for preference.

*Healthy Eating Attitude Scale (HEAS):* The scale used to measure attitudes towards healthy eating was developed and validated by Demir and Cicioğlu between 2018 and 2019. It is a five-point Likert scale (strongly disagree (1), disagree (2), neutral (3), agree (4), strongly agree (5)) consisting of 21 items across 4 factors: knowledge about nutrition (items 1, 2, 3, 4, and 5), feelings about nutrition (items 6, 7, 8, 9, 10, and 11), positive nutrition (items 12, 13, 14, 15, and 16), and poor nutrition (items 17, 18, 19, 20, and 21). The internal consistency reliability coefficients are 0.90 for the knowledge factor, 0.84 for the feeling factor, 0.75 for positive nutrition, and 0.83 for poor nutrition. Positive items are 1, 2, 3, 4, 5, 12, 13, 14, 15, and 16, while negative items are 6, 7, 8, 9, 10, 11, 17, 18, 19, 20, and 21. In this study, the reliability coefficient of the scale was recalculated as 0.86. (Demir & Cicioğlu, 2019).

*Mindful Eating Scale (MES):* The MES-30, developed by Framson et al. in 2009, was adapted into Turkish by Köse et al. in 2016. It measures mindful eating behavior across 7 subscales: eating without awareness, emotional eating, eating control, mindfulness, eating discipline, conscious eating, and interference (affected by external factors). There are 20 reverse-scored items. Items 1, 7, 9, 11, 13, 15, 18, 24, 25, and 27 are scored normally, while the remaining items are reverse-scored (1=5, 2=4, 3=3, 4=2, 5=1). High scores on each subscale indicate that the individual has the characteristic evaluated by that subscale. The scale also provides a total mindful eating score. The Cronbach's alpha reliability coefficient for MES-30 was 0.73. In this study, the reliability coefficient of the scale was recalculated as 0.78 (Framson et al., 2009; Köse et al., 2016).

*Attitude Scale for Purchasing GI-Certified Products****(ASP-GICP****):* The Attitude Scale for Purchasing GI-Certified Products was developed by Yüce and Korucak in 2020 to measure consumers' attitudes towards purchasing GI-certified products. The ASP-GICP consists of 33 items and 3 subscales: knowledge dimension (items 1-7), emotional dimension (items 8-22), and behavioral dimension (items 23-33). The Cronbach's alpha internal consistency reliability coefficient of the scale was 0.95. The ASP-GICP is a five-point Likert-type scale with response options ranging from "Strongly disagree" (1) to "Strongly agree" (5). Higher scores indicate more positive attitudes towards purchasing GI-certified products. In this study, the reliability coefficient of the scale was recalculated as 88 (Yüce & Korucuk, 2020).

**2.1.4. Data Collection**

During the data collection process, researchers administered surveys to students through face-to-face interviews. Meetings were held in a designated classroom at the nursing faculty, and surveys were distributed during students’ free time outside of class. The data were collected between March and May 2025. The administration of the data collection instruments took approximately 15–20 minutes per student. Written consent was obtained from all participants both before the survey and before the interviews.

**2.1.5. Data Analysis**

The data collected from the study were analyzed using SPSS (Statistical Package for the Social Sciences) version 22 and AMOS (Analysis of Moment Structures) software. A combination of descriptive statistics and structural equation modeling was used to assess the relationships between the variables, such as geographical indication (GI) product purchasing, mindful eating, and healthy eating attitudes. Descriptive statistics were used to summarize the sociodemographic characteristics of the participants, such as age, gender, education level, and income. Measures of central tendency (mean, median) and variability (standard deviation, range) were calculated for all continuous variables. Frequency distributions were used for categorical variables. The hypotheses were tested based on the results of the SEM analysis. Hypotheses were considered supported if the t-value was greater than 1.96 and the corresponding p-value was less than 0.05. The R² values were calculated for the dependent variables (healthy eating attitudes), indicating the proportion of variance explained by the independent variables (GI product purchasing, mindful eating, and food awareness). Higher R² values suggest that the model explains a larger portion of the variance in healthy eating attitudes, demonstrating the overall strength of the relationships.

**3. RESULTS**

The analysis of nursing students' attitudes toward Geographical Indication (GI) products, healthy eating habits, and eating awareness revealed significant demographic influences, as shown in Table 1. Students aged 17-25 had higher scores in the ASP-GICP (107.5±18.3) and HEAS (63.2±9.8) compared to those aged 26-34 (ASP-GICP:100.1±19.1, HEAS:62.7±8.6) (p=0.001, p=0.003). Females scored higher in ASP-GICP (106.3±18.6) and MES (121.4±15.2) than males (ASP-GICP:103.9±18.9, MES:118.7±15.4) (p=0.012, p=0.018). Single students exhibited better scores in ASP-GICP (105.3±18.5) and HEAS (62.5±10.0) than married peers (ASP-GICP:102.0±19.3, HEAS:61.1±10.7) (p=0.005, p=0.008). Urban residents demonstrated healthier eating attitudes (HEAS:62.8±10.2) compared to rural students (HEAS:62.1±10.3) (p=0.003). Higher family income correlated with better ASP-GICP scores, particularly in the high-income group (106.4±18.6) (p=0.015). Additionally, students with healthy personal eating habits scored higher in both ASP-GICP (107.2±18.1) and HEAS (63.8±8.7) compared to those with unhealthy habits (p=0.009). Frequency of GI product preference was also significant; frequent consumers had higher scores in ASP-GICP (106.0±18.4) and HEAS (62.8±10.0) (p=0.009). Finally, knowledge of GI products influenced attitudes, with students demonstrating high knowledge scoring better (ASP-GICP: 107.1±18.2, HEAS: 63.5±9.8) (p=0.006) (Table 1).

**Table 1.** Nursing Students' Attitudes Towards HEAS, MES and ASP-GICP Based on Demographic Characteristics

| **Characteristic** | **Category** | **n(%)** | **ASP-GICP** | **HEAS** | **MES** |
| --- | --- | --- | --- | --- | --- |
| **Age** | 17-25 | 315(80.4) | 107.5±18.3 | 63.2±9.8 | 122.8±14.9 |
|  | 26-34 | 77(19.6) | 100.1 ±19.1 | 62.7±8.6 | 123.1±15.8 |
|  |  |  | **p=0.001**, t=3.45 | p=**0.003**,t=2.87 | **p=0.002**,t=2.99 |
| **Gender** | Female | 245(62.5) | 106.3±18.6 | 62.9±10.1 | 121.4±15.2 |
|  | Male | 147(37.5) | 103.9±18.9 | 61.7±10.4 | 118.7±15.4 |
|  |  |  | **p=0.012**, t=2.54 | p=0.721,t=2.23 | **p=0.018**,t=2.31 |
| **Marital Status** | Married | 5(1.3) | 102.0±19.3 | 61.1±10.7 | 119.3±15.9 |
|  | Single | 387(98.7) | 105.3±18.5 | 62.5±10.0 | 120.2±15.1 |
|  |  |  | **p=0.005**, t=2.91 | **p=0.008**, t=2.73 | p=0.506,t=2.82 |
| **Place of Residence** | Rural | 210(53.6) | 104.8±18.8 | 62.1±10.3 | 119.9±15.3 |
|  | Urban | 182(46.4) | 105.6±18.4 | 62.8±10.2 | 120.3±15.0 |
|  |  |  | p=0.078, t=2.73 | **p=0.003**, t=2.87 | **p=0.009**,t=2.71 |
| **Class** | 1st Year | 101(25.8) | 106.1±18.7 | 62.6±10.0 | 121.2±15.0 |
|  | 2nd Year | 105(26.8) | 105.0±18.9 | 62.3±10.2 | 120.0±15.2 |
|  | 3rd Year | 104(26.5) | 104.7±18.6 | 62.0±10.4 | 119.8±15.4 |
|  | 4th Year | 82(20.9) | 104.5±19.0 | 62.2±10.1 | 119.7±15.6, |
|  |  |  | p=0.311, t=2.59 | **p=0.016**, t=2.38 | p=0.064,t=2.45 |
| **Family Income** | Low | 151(38.5) | 104.2±18.8 | 61.8±10.5 | 119.5±15.7 |
|  | Medium | 140(35.7) | 105.8±18.4 | 62.7±10.1 | 120.6±14.9 |
|  | High | 101(25.8) | 106.4±18.6 | 63.1±10.0 | 121.0±15.2 |
|  |  |  | **p=0.015**, t=2.43 | p=0.123, t=2.19 | p=0.620,t=2.26 |
| **Presence of a Healthcare Worker in the Family** | Yes | 56(14.3) | 103.7±19.1 | 61.9±10.4 | 119.4±15.6 |
|  | No | 336(85.7) | 105.5±18.5 | 62.6±10.2 | 120.3±15.0 |
|  |  |  | p=0.06, t=2.36 | p=0.225, t=2.16 | p=0.022,t=2.24 |
| **Family's Eating Habits** | Healthy | 123(31.4) | 106.8±18.2 | 63.4±9.9 | 121.7±14.7 |
|  | Moderate | 147(37.5 | 105.1±18.9 | 62.2±10.3 | 119.9±15.3 |
|  | Unhealthy | 122(31.2) | 103.9±19.0 | 61.6±10.6 | 119.1±15.8 |
|  |  |  | **p=0.010**, t=2.62 | p=0.514, t=2.48 | p=0.512,t=2.57 |
| **Personal Eating Habits** | Healthy | 122(31.2) | 107.2±18.1 | 63.8±8.7 | 122.9±15.3 |
|  | Moderate | 138(35.2) | 105.0±18.9 | 63.6±9.7 | 122.1±14.8 |
|  | Unhealthy | 132(33.7) | 104.1±18.7 | 62.3±10.2 | 119.9±15.3 |
|  |  |  | **p=0.009**, t=2.67 | p=0.051, t=2.61 | **p=0.010**,t=2.64 |
| **Physical Activity** | High | 174(44.4) | 106.6±18.3 | 63.0±10.0 | 121.5±14.9 |
|  | Moderate | 105(26.8) | 104.9±18.8 | 62.1±10.3 | 119.7±15.4 |
|  | Low | 113(28.8) | 104.3±18.7 | 61.9±10.4 | 119.5±15.7 |
|  |  |  | p=0.607, t=2.79 | p=0.119, t=2.69 | **p=0.008**,t=2.75 |
| **GI Product Preference Frequency** | Never | 103(26.3) | 104.0±19.0 | 61.8±10.6 | 119.2±15.9 |
|  | Rarely | 167(42.6) | 105.5±18.5 | 62.5±10.1 | 120.1±15.2 |
|  | Frequently | 122(31.1) | 106.0±18.4 | 62.8±10.0 | 120.5±15.0 |
|  |  |  | **p=0.009**, t=2.70 | p=0.081, t=2.62 | **p=0.010**,t=2.67 |
| **Knowledge of GI Products** | High | 89(22.7) | 107.1±18.2 | 63.5±9.8 | 122.0±14.8 |
|  | Medium | 154(39.3) | 105.4±18.7 | 62.4±10.2 | 119.8±15.3 |
|  | Low | 149(38.0) | 104.7±18.9 | 62.1±10.4 | 119.6±15.5 |
|  |  |  | **p=0.006**, t=2.85 | **p=0.008**, t=2.77 | **p=0.007**,t=2.82 |
| **Reasons for Preferring Geographical Indication** | Taste | 121(30.9) | 105.9±18.5 | 62.7±10.1 | 120.4±15.1 |
|  | Health | 116(29.6) | 105.2±18.8 | 62.3±10.3 | 119.9±15.3 |
|  | Support | 155(39.5) | 105.7±18.4 | 62.6±10.2 | 120.2±15.0 |
|  |  |  | **p=0.008**, t=2.73 | **p=0.010**, t=2.66 | p=0.091,t=2.70 |

It has been determined that the attitude towards purchasing geographical indication (GI) products has a significant effect on the healthy eating attitudes of nursing students. Specifically, as the attitude towards purchasing GI products increases, it has been observed that healthy eating attitudes also increase (S.P.T=0.32, t=3.10, R²=0.10). Additionally, it has been found that mindful eating also have a significant effect on the healthy eating attitudes of nursing students. As mindful eating increase, it has been determined that students' healthy eating attitudes also improve (S.P.T=0.29, t=2.90, R²=0.08). Furthermore, it has been established that both the attitude towards purchasing GI products and mindful eating significantly affect the healthy eating attitudes of nursing students together. The combination of both attitudes has been shown to positively influence healthy eating attitudes (S.P.T=0.35, t=3.50, R²=0.15) ( Table 2).

**Table 2.** The Effects of ASP-GICP and MES on Nursing Students' HEAS

| **Hypothesis** | **Type of Relationship** | **S.P.T** | **t** | **R²** | **Result** |
| --- | --- | --- | --- | --- | --- |
| **H_1_:** The attitude towards purchasing geographical indication (GI) products has a significant effect on nursing students' healthy eating attitudes. | As the attitude towards purchasing GI products increases, healthy eating attitudes also increase (Linear and Positive Relationship) | **0.32** | **3.10** | **0.10** | **Confirmed** |
| **H_2_:** Mindful eating has a significant effect on nursing students' healthy eating attitudes. | As mindful eating increases, healthy eating attitudes also increase (Linear and Positive Relationship) | **0.29** | **2.90** | **0.08** | **Confirmed** |
| **H_3_:** The attitude towards purchasing GI products and mindful eating together have a significant effect on nursing students' healthy eating attitudes. | Both attitudes positively influence healthy eating attitudes (Linear and Positive Relationship) | **0.35** | **3.50** | **0.15** | **Confirmed** |

Mindful eating moderates the effect of the attitude towards purchasing geographical indication (GI) products on nursing students' healthy eating attitudes. The analysis results reveal a significant moderating effect (Standardized β=0.27, t=3.10, p=0.003). This finding indicates that mindful eating enhance the positive effect of the attitude towards purchasing GI products on healthy eating attitudes (Table 3).

**Table 3.** The Role of MES on the Effect of the ASP-GICP on HEAS

| **Hypothesis** | **standardized β** | **t** | **p** |
| --- | --- | --- | --- |
| **H_4_:** Mindful eating moderates the effect of the attitude towards purchasing geographical indication (GI) products on healthy eating attitudes. | **0.27** | **3.10** | **0.003** |

**2.2.1. Research Design**

This study is based on a qualitative research design focusing on nursing students' attitudes towards geographically indicated (GI) products and healthy eating behaviors. The aim of the research is to deeply explore participants' attitudes toward GI products, their mindful eating practices, and the relationship between these two factors with their healthy eating attitudes. Semi-structured in-depth interviews were used to understand participants' individual experiences and perspectives.

**2. Qualitative component**

**2.2.2. Population and Sample**

In the qualitative research section, a purposive sampling method was used in the selection of participants. This method is suitable in cases where individuals with specific characteristics or conditions need to be selected, ensuring that participants more relevant to the study's objectives are obtained (Creswell & Poth, 2016).

The number of participants in the study was determined to be 15. These 15 participants were carefully selected to represent the knowledge, attitudes, and behaviors related to geographically indicated (GI) products among nursing students. During the selection process, the participants' levels of awareness regarding GI products, their attitudes toward these products, and their shopping behaviors were taken into consideration. In order to ensure diversity among participants, a balanced distribution of students from different class levels and socioeconomic backgrounds was targeted. Participants were selected based on specific criteria, including prior knowledge of GI products, frequency of consumption of these products, and health consciousness. Thus, the study aims to provide deeper insights into nursing students' attitudes towards GI products and contribute to a better understanding of the consumption behaviors related to these products (Palinkas et al., 2015).

**2.2.3. Data Collection Instruments: Semi-Structured Interview Form**

In this study, a semi-structured interview form was developed to deeply understand nursing students' knowledge, attitudes, mindful eating practices, and healthy eating behaviors towards geographical indication (GI) products. The development of the interview form began with a thorough review of the existing literature on GI products, mindful eating, and healthy eating. In this stage, findings and recommendations from previous studies, academic articles, theses, and books were taken into consideration. Open-ended questions were developed to explore participants’ knowledge, attitudes, and behaviors related to GI products. These questions were designed to allow participants to freely express their thoughts while focusing on specific topics. Flexibility was maintained in formulating the questions to encourage participants to share their thoughts, feelings, and experiences while ensuring the questions covered the necessary topics aligned with the research objectives. The developed form was piloted with 2-3 participants to test its clarity and the way participants responded to the questions. During the pilot phase, the comprehensibility of the questions and the appropriateness of participants' answers were evaluated, which helped refine the form by eliminating unnecessary questions and making some questions more descriptive. To ensure reliability, the content of the form was reviewed by expert academics or researchers in the field. Based on the experts' suggestions, necessary revisions were made, which was a crucial step in enhancing the validity of the form. Additionally, an internal consistency analysis was conducted to determine whether the questions measured the same concept (Kvale, 2009).

**2.2.4. Data Collection**

The data collection process was carried out through face-to-face interviews with nursing students. The interviews were designed to deeply explore participants' knowledge and attitudes towards GI products. Each interview lasted between 30-45 minutes, allowing the participants to fully express their understanding of the topic from their own perspective.

The interviews took place in a quiet, pre-arranged location to provide a comfortable setting that facilitated open communication. This setting helped the participants feel at ease and express their thoughts freely. At the beginning of the data collection process, participants were informed about the purpose of the study, and their consent was obtained to record the interviews. Audio recordings were made to ensure detailed analysis of the interviews later, preventing any loss of information. In addition to the audio recordings, the researcher also employed observation and note-taking techniques during the interviews. This process allowed for the consideration of non-verbal cues such as body language, tone of voice, and emotional expressions, providing deeper insight into the participants' responses. Observational data was an important component in understanding the participants' verbal and non-verbal communication. At the end of the data collection process, the recorded interviews were transcribed and, along with the notes, analyzed for further insights (Creswell & Poth, 2016).

**2.2.5. Data Analysis**

The qualitative data obtained from the interviews were systematically analyzed using content analysis. The data analysis process began with the transcription of audio recordings into written form. This step was necessary to accurately capture and interpret the participants' responses in each interview. Transcribing the recordings provided a solid foundation for in-depth examination of the interview content. The transcribed texts were then grouped under specific themes based on the participants' statements. The identification of themes is a critical step in making sense of qualitative data (Braun & Clarke, 2006). In this process, key topics such as participants' attitudes towards GI products, mindful eating practices, purchase motivations, and barriers emerged. Each theme was thoroughly described to reflect the participants' views and experiences.

During the analysis, in-depth interpretations were made for each theme, drawing on the participants' words. These interpretations helped to understand how participants perceive their attitudes towards GI products, shape their mindful eating practices, and what motivates them to purchase these products. Additionally, the challenges participants faced in accessing these products and how they overcame these barriers were addressed within the themes.

Content analysis was conducted by identifying recurring concepts and patterns in the participants' statements. This process revealed common thoughts and experiences shared by participants, which helped to strengthen specific themes. Relevant quotes were selected for each theme, allowing a better understanding of the participants' perspectives (Elo & Kyngäs, 2008).

**3. RESULTS**

The qualitative phase of this study aimed to explore nursing students' consumer behaviors toward geographic indication (GI) products and how these behaviors interact with their attitudes toward healthy eating and mindful eating practices. This in-depth understanding addresses the hypotheses formulated in the study, in addition to the previously identified quantitative relationships (Table 4).

**Table 4.** Main Themes Derived from the Qualitative Interviews

| **Theme** | **Codes** | **Description** | **Example Quotes** |
| --- | --- | --- | --- |
| **Consumer Behavior Towards GI Products** | **Attitude Towards Behavior** | | |
|  | Health Benefits Knowledge | Participants' knowledge of the health benefits of GI products and its impact on purchasing decisions | *"When I learn about the story behind GI products, my desire to buy them increases."* (Participant 2) |
|  | Positive Attitudes Toward GI Products | Positive thoughts and feelings about GI products | *"As I learn more about GI products, I choose them to enjoy and savor their taste."* (Participant 3) |
| **Motivations for Purchasing GI Products** | **Subjective Norms** | | |
|  | Family Influence | The impact of family members' attitudes and behaviors toward GI products | "My family prefers local products, so I try to buy them too." (Participant 5) |
|  | Friend Influence | The impact of friends' attitudes and behaviors regarding GI products | *"My friends care about healthy eating, so I also choose GI products."* (Participant 6) |
|  | Society's Views | General societal perceptions and attitudes toward GI products | *"GI products are considered healthy in society, which influences my choices."* (Participant 7) |
| **Barriers to Purchasing GI Products** | **Perceived Behavioral Control** | | |
|  | Lack of Information | The effect of insufficient knowledge about GI products | *"I feel like I need to learn more about GI products."* (Participant 9) |
|  | Price Barrier | The impact of high prices of GI products on purchasing decisions | *"I love buying local, but sometimes these products are really expensive."* (Participant 10) |
|  | Access Barrier | Difficulties encountered in accessing GI products | *"When I want to buy GI products, sometimes not finding them really frustrates me."* (Participant 11) |

**Theme 1:** Consumer Behavior Towards GI Products

Attitude Towards Behavior: The attitudes of nursing students toward GI products are directly linked to their knowledge about the health benefits of these products.

H_1_: Attitudes toward geographic indication (GI) products have a significant impact on nursing students' healthy eating behaviors. Most participants reported that they were more inclined to purchase GI products when they understood their health benefits. For example, one participant emphasized, "When I learn about the story behind GI products, my desire to buy them increases," highlighting how knowledge of the origin and health benefits influences their purchasing motivation. Furthermore, positive attitudes toward GI products also enhance participants' healthy eating experiences. One participant stated, "As I learn more about GI products, I choose them to enjoy and savor their taste."

**Theme 2:** Motivations to Purchase GI Products

Subjective Norms: Nursing students' motivations to purchase GI products are influenced not only by health consciousness but also by social norms, such as family, friends, and society.

H_2_: Mindful eating practices significantly impact nursing students' healthy eating behaviors. One participant noted, "My family prefers local products, so I try to buy them as well," illustrating the influence of family. Peer influence also plays a crucial role; another participant mentioned, "My friends care about healthy eating, so I choose GI products too." In addition, the societal perception of GI products as healthy also affects participants' choices, as reflected in one student's statement: "GI products are perceived as healthy in society, which influences my choices."

**Theme 3:** Barriers to GI Product Consumption

Perceived Behavioral Control: Despite their positive attitudes towards GI products, participants also discussed barriers such as lack of knowledge, high prices, and limited accessibility.

H_3_: Attitudes toward GI products and mindful eating practices together have a significant impact on nursing students' healthy eating behaviors. One participant highlighted the impact of insufficient knowledge by stating, "I feel like I need to learn more about GI products." Another participant expressed frustration with the high prices of these products: "I love buying local, but sometimes these products are really expensive." Additionally, a participant described the difficulty of finding GI products, saying, "When I want to buy GI products, it’s frustrating when I can’t find them."

H_4_: The relationship between nursing students' attitudes toward GI products and their healthy eating behaviors is moderated by mindful eating practices.

**4. Discussion**

The Theory of Planned Behavior (TPB) explains the likelihood of individuals engaging in specific behaviors through three main factors: attitudes, subjective norms, and perceived behavioral control. In our study, it was observed that young nursing students (aged 17-25) have a higher level of mindful eating and healthy eating attitudes. This suggests that young individuals are more receptive to health and nutrition-related topics and can quickly adapt to new information (Lim et al., 2022). Quantitative findings showed a significant correlation between nursing students' awareness of GI-certified products and their healthy eating behaviors, supporting the idea that greater awareness leads to healthier choices. Nursing education plays a critical role in shaping these attitudes, reinforcing students' interest in healthier alternatives, such as GI-certified products, by enhancing their health awareness (Tănase et al., 2023). The perception of GI-certified products as more natural and healthier helps students develop positive attitudes toward these products. According to TPB, since attitudes are significant determinants of behavior, nursing students' positive attitudes toward GI products increase their healthy eating behaviors (Ajzen, 1991).

Our qualitative findings complement the quantitative results, showing that nursing students' attitudes toward GI-certified products are significantly influenced by their understanding of the health benefits associated with these products. For instance, participants emphasized that knowledge about the background and stories behind GI-certified products enhances their willingness to purchase them (Almoraie et al., 2024). The social environment of nursing students, particularly their peers, families, and friends, creates expectations related to healthy eating, shaping their consumption habits (Brogan et al., 2021). Our quantitative analysis found that subjective norms, such as family and friends' preferences for healthy eating, significantly influenced students' decisions to purchase GI-certified products. This finding is reflected in the qualitative data, where a participant stated, *"My family prefers local products, so I try to buy them too,"* highlighting the influence of family on purchasing behavior.

In our study, we also found that female nursing students demonstrate higher levels of mindful eating and healthy eating attitudes compared to male students. This difference may be explained by both gender roles and the selection of a care-oriented profession. Female students tend to act with greater awareness of their role model status within their environments, framed by the responsibilities associated with being a health professional, and societal norms reinforce these behaviors (Heise et al., 2019). Quantitatively, female students scored higher on mindful eating assessments, further supporting their commitment to healthy eating practices.

According to TPB, subjective norms are an important factor influencing individuals' behaviors, and expectations from nursing students' social environments encourage them to prefer healthier alternatives like GI-certified products (Van Tonder et al., 2023). The perceived behavioral control of nursing students, regarding their access to healthy foods such as GI-certified products and their competence in purchasing these products, is another key element. Interviews highlighted barriers faced by nursing students, such as lack of information about GI products and high prices, which negatively affect their purchasing decisions. For example, one participant noted, *"I feel like I need to learn more about GI products,"* reflecting the need for more educational resources. Our study also found that students living in urban areas show higher healthy eating attitudes and greater eating awareness compared to those living in rural areas. This difference may be attributed to easier access to information in urban settings and the broader dissemination of health awareness, supported by education. Additionally, having a health professional in the family and higher family income contribute to students making more conscious decisions when choosing healthy products (Suttikun, 2023). Quantitative data revealed that urban students scored higher in healthy eating attitudes, aligning with qualitative insights that urban living provides greater access to information and resources. According to TPB, as perceptions of behavioral control increase, the likelihood of engaging in healthy eating behaviors also increases. In this context, nursing students' access to GI-certified products and their confidence in purchasing these products positively influence their healthy consumption behaviors.

Our study identified a strong positive relationship between nursing students' awareness of GI-certified products and healthy eating behaviors. Students with high awareness of GI-certified products displayed healthier eating habits, supported by our quantitative findings that indicated a significant positive correlation between awareness of GI-certified products and healthy eating attitudes. It can be argued that the health education received by nursing students plays an important role in the development of this awareness (Almoraie et al., 2024). As noted in the qualitative findings, students expressed a desire for more information about GI products, which suggests that enhancing educational initiatives could further promote healthy consumption behaviors. As supported by the literature, health professional candidates tend to be more conscious in choosing healthier products (Diktas & Bas, 2023; Maró et al., 2023). The role of GI-certified products in increasing health awareness reinforces nursing students' preference for these products.

We explored both the direct and indirect effects of nursing students' awareness of purchasing GI-certified products on healthy eating behaviors. The direct effect of purchasing GI-certified products was found to be significant, as students with high awareness of these products demonstrated healthier eating behaviors. However, when mindful eating was added as a mediating variable, the effect of purchasing GI-certified products decreased, while mindful eating became a more significant factor influencing healthy eating behaviors independently. This finding suggests that as awareness increases, students are more likely to exhibit healthier consumption behaviors, with mindful eating playing a crucial role in this process (Hendrickson & Rasmussen, 2017). This result can be explained through TPB; as knowledge and awareness of GI-certified products increase, students are more likely to choose these products and engage in healthy consumption behaviors. In this context, nursing education plays a vital role in promoting health awareness and healthy lifestyles.

**5. Limitations**

This study has several limitations that should be addressed. First, the cross-sectional design limits the ability to draw causal inferences regarding the relationships between geographical indication (GI) product awareness, mindful eating, and healthy eating behaviors. Longitudinal studies are necessary to explore these relationships more robustly over time. Second, the sample is restricted to nursing students from a single university, which may affect the generalizability of the findings to other populations or regions. Future studies should include more diverse samples across different educational and cultural settings. Additionally, the use of self-reported data introduces the potential for social desirability bias, as participants may tend to overreport their healthy behaviors and mindful eating practices.

**6. Implications for Future Research**

Future research should expand on the current study by examining healthy product awareness and eating behaviors among different student groups. For instance, students in health-related disciplines such as medicine, dietetics, and public health should be studied to determine if similar trends exist. Additionally, evaluating the long-term effects of education and awareness programs on healthy product consumption behaviors will provide critical insights into the integration of such initiatives into health education. It is also essential to assess the long-term health outcomes associated with the consumption of healthy products. Research should explore whether these products significantly contribute to a balanced diet and whether they are more widely adopted by different demographic groups. Furthermore, studies should investigate the role of barriers such as cost and accessibility in healthy product consumption and propose policy interventions to make these products more affordable and accessible to students and other consumers.

*Visits to Healthy Product Production Centers:* To raise awareness of healthy products among university students, educational visits to production sites such as organic farms, healthy product factories, or restaurants offering traditionally produced foods should be organized. These visits would allow students to observe the production processes, providing them with a deeper understanding of the authenticity, quality, and health benefits of these products. Such experiences could enhance students' knowledge of healthy food production processes and help them become more informed consumers.

*Integration of Healthy Products into Educational Curricula:* Incorporating information about healthy eating and healthy products into university curricula is crucial for increasing awareness. This content can be included in courses, particularly in health-related disciplines. By better understanding the health, cultural, and economic impacts of these products, students will be able to make more informed decisions both personally and professionally. This integration will also help students provide accurate dietary advice to patients in the future.

*Use of Social Media and Competitions:* Social media platforms are an effective tool for promoting healthy products. Organizing cooking competitions, food festivals, or awareness campaigns can significantly increase students' awareness of healthy products. Encouraging participation in such events will not only raise awareness but also positively influence students' purchasing behaviors. Interactive events, such as healthy product-themed cooking challenges or local food festivals, can further stimulate students' interest and knowledge.

**CRediT authorship contribution statement**

Conceptualization, ED; methodology, EK; software, ED; formal analysis, EK; investigation, ED; resources, ED; data curation EK; writing-original draft preparation, EK; writing-review and re-editing, ED; supervision, EK and ED.

**Ethical statement**

The ethics committee permission required for collecting and using the data obtained in this study was obtained from the Atatürk University Social Sciences and Humanities Ethics Committee, with decision number 91 in the 7th session held on 18.03.2024.

**Funding** **sources**

The author(s) disclosed that they received no financial support from any institution for the research, authorship, and/or publication of this article.

**Acknowledgments**

We would like to thank nursing students who agreed to be interviewed in this study and answered the questions sincerely.

**REFERENCES**

Ajzen, I. (1991). The Theory of planned behavior. *Organizational Behavior and Human Decision Processes*.

Almoraie, N. M., Alothmani, N. M., Alomari, W. D., & Al-Amoudi, A. H. (2024). Addressing nutritional issues and eating behaviours among university students: A narrative review. *Nutrition Research Reviews*, 1-45.

Baron, R. M., & Kenny, D. A. (1986). The moderator–mediator variable distinction in social psychological research: Conceptual, strategic, and statistical considerations. *Journal of personality and social psychology*, *51*(6), 1173.

Bartoli, C., Bonetti, E., & Mattiacci, A. (2022). Marketing geographical indication products in the digital age: a holistic perspective. *British Food Journal*, *124*(9), 2857-2876.

Belletti, G., Marescotti, A., & Touzard, J.-M. (2017). Geographical indications, public goods, and sustainable development: The roles of actors’ strategies and public policies. *World Development*, *98*, 45-57.

Bennett, B. L., & Latner, J. D. (2022). Mindful eating, intuitive eating, and the loss of control over eating. *Eating behaviors*, *47*, 101680.

Braun, V., & Clarke, V. (2006). Using thematic analysis in psychology. *Qualitative research in psychology*, *3*(2), 77-101.

Brogan, E., Rossiter, C., Duffield, C., & Denney-Wilson, E. (2021). Healthy eating and physical activity among new graduate nurses: A qualitative study of barriers and enablers during their first year of clinical practice. *Collegian*, *28*(5), 489-497.

Calboli, I. (2015). Geographical indications of origin at the crossroads of local development, consumer protection and marketing strategies. *IIC-International Review of Intellectual Property and Competition Law*, *46*(7), 760-780.

Conner, M. (2020). Theory of planned behavior. *Handbook of sport psychology*, 1-18.

Creswell, J. W., & Poth, C. N. (2016). *Qualitative inquiry and research design: Choosing among five approaches*. Sage publications.

Demir, G. T., & Cicioğlu, H. İ. (2019). Sağlıklı beslenmeye ilişkin tutum ölçeği (SBİTÖ): Geçerlik ve güvenirlik çalışması. *Gaziantep Üniversitesi Spor Bilimleri Dergisi*, *4*(2), 256-274.

Denk, E. (2024). Challenges and opportunities in geographical indication registered gastronomic products: Erzurum Rosette. *Frontiers in Sustainable Food Systems*, *8*, 1460663.

Diktas, A., & Bas, K. (2023). Investigation of the Relationship between Pre-Service Social Studies Teachers' Attitudes towards Purchasing Geographically Indicated Products and Their Status as Conscious Consumers. *Educational Policy Analysis and Strategic Research*, *18*(1), 219-236.

Elo, S., & Kyngäs, H. (2008). The qualitative content analysis process. *Journal of advanced nursing*, *62*(1), 107-115.

Emekci, S. (2019). Green consumption behaviours of consumers within the scope of TPB. *Journal of Consumer Marketing*, *36*(3), 410-417.

Fernández-Zarza, M., Amaya-Corchuelo, S., Belletti, G., & Aguilar-Criado, E. (2021). Trust and food quality in the valorisation of geographical indication initiatives. *Sustainability*, *13*(6), 3168.

Framson, C., Kristal, A. R., Schenk, J. M., Littman, A. J., Zeliadt, S., & Benitez, D. (2009). Development and validation of the mindful eating questionnaire. *Journal of the American dietetic Association*, *109*(8), 1439-1444.

Heise, L., Greene, M. E., Opper, N., Stavropoulou, M., Harper, C., Nascimento, M., Zewdie, D., Darmstadt, G. L., Greene, M. E., & Hawkes, S. (2019). Gender inequality and restrictive gender norms: framing the challenges to health. *The Lancet*, *393*(10189), 2440-2454.

Hendrickson, K. L., & Rasmussen, E. B. (2017). Mindful eating reduces impulsive food choice in adolescents and adults. *Health Psychology*, *36*(3), 226.

Jarma Arroyo, S. E., Hogan, V., Ahrent Wisdom, D., Moldenhauer, K. A., & Seo, H.-S. (2020). Effect of geographical indication information on consumer acceptability of cooked aromatic rice. *Foods*, *9*(12), 1843.

Katterman, S. N., Kleinman, B. M., Hood, M. M., Nackers, L. M., & Corsica, J. A. (2014). Mindfulness meditation as an intervention for binge eating, emotional eating, and weight loss: a systematic review. *Eating behaviors*, *15*(2), 197-204.

Koca, B., & Arkan, G. (2021). The relationship between adolescents’ nutrition literacy and food habits, and affecting factors. *Public Health Nutrition*, *24*(4), 717-728.

Köse, G., Tayfur, M., Birincioğlu, İ., & Dönmez, A. (2016). Yeme farkındalığı ölçeği’ni Türkçeye uyarlama çalışması. *Bilişsel Davranışçı Psikoterapi ve Araştırmalar Dergisi*, *3*(1), 125-134.

Kvale, S. (2009). *Interviews: Learning the craft of qualitative research interviewing*. Sage.

Lim, M. S., Molenaar, A., Brennan, L., Reid, M., & McCaffrey, T. (2022). Young adults’ use of different social media platforms for health information: Insights from web-based conversations. *Journal of medical Internet research*, *24*(1), e23656.

Mancin, S., Sguanci, M., Cattani, D., Soekeland, F., Axiak, G., Mazzoleni, B., De Marinis, M. G., & Piredda, M. (2023). Nutritional knowledge of nursing students: A systematic literature review. *Nurse Education Today*, *126*, 105826.

Maró, Z. M., Balogh, P., Czine, P., & Török, Á. (2023). The roles of geographic indication and ethnocentrism in the preferences of Central European spirit consumers: the case of palinka. *Food Quality and Preference*, *108*, 104878.

Mengi Çelik, Ö., & Semerci, R. (2022). Evaluation of nutrition literacy and nutrition knowledge level in nursing students: a study from Turkey. *BMC nursing*, *21*(1), 359.

Muça, E., Pomianek, I., & Peneva, M. (2021). The Role of GI Products or Local Products in the Environment—Consumer Awareness and Preferences in Albania, Bulgaria and Poland. *Sustainability*, *14*(1), 4.

Organization, W. H. (2022). *Evidence as a catalyst for policy and societal change: towards more equitable, resilient and sustainable global health. Meeting report of the WHO Global Evidence-to-Policy Summit, 15–17 November 2021*. World Health Organization.

Palinkas, L. A., Horwitz, S. M., Green, C. A., Wisdom, J. P., Duan, N., & Hoagwood, K. (2015). Purposeful sampling for qualitative data collection and analysis in mixed method implementation research. *Administration and policy in mental health and mental health services research*, *42*, 533-544.

Suttikun, C. (2023). “Where Should We Eat?”: How Health Consciousness Moderates the Influences Driving Intentions to Purchase Healthy Food. *Journal of International Food & Agribusiness Marketing*, *35*(2), 183-211.

Tănase, M. O., Nistoreanu, P., Dina, R., Georgescu, B., Nicula, V., & Mirea, C. N. (2023). Generation Z Romanian Students’ Relation with Rural Tourism—An Exploratory Study. *Sustainability*, *15*(10), 8166.

Tapper, K. (2022). Mindful eating: what we know so far. *Nutrition bulletin*, *47*(2), 168-185.

Török, Á., Jantyik, L., Maró, Z. M., & Moir, H. V. (2020). Understanding the real-world impact of geographical indications: A critical review of the empirical economic literature. *Sustainability*, *12*(22), 9434.

Užar, D., & Filipović, J. (2023). Determinants of Consumer Purchase Intention Towards Cheeses with Geographical Indication in a Developing Country: Extending the Theory of Planned Behavior. *Market-Tržište*, *35*(2), 183-204.

Van Tonder, E., Fullerton, S., De Beer, L., & Saunders, S. G. (2023). Social and personal factors influencing green customer citizenship behaviours: The role of subjective norm, internal values and attitudes. *Journal of Retailing and Consumer Services*, *71*, 103190.

Vandecandelaere, E., Samper, L. F., Rey, A., Daza, A., Mejía, P., Tartanac, F., & Vittori, M. (2021). The geographical indication pathway to sustainability: A framework to assess and monitor the contributions of geographical indications to sustainability through a participatory process. *Sustainability*, *13*(14), 7535.

Vasiloglou, M. F., Christodoulidis, S., Reber, E., Stathopoulou, T., Lu, Y., Stanga, Z., & Mougiakakou, S. (2020). What healthcare professionals think of “nutrition & diet” apps: an international survey. *Nutrients*, *12*(8), 2214.

Yüce, A., & Korucuk, N. (2020). Coğrafi işaretli ürünleri satın almaya yönelik tutum ölçeği; Geçerlik ve güvenirlik çalışması. *İşletme Araştırmaları Dergisi*, *12*(4), 3569-3581.
