# Supplementary material for: Geographical indications and health-conscious behaviors among nursing students: A mixed methods study
Source: PLoS One. 2026 Mar 5;21(3):e0344242. doi: 10.1371/journal.pone.0344242 (PMC12962537; doi:10.1371/journal.pone.0344242)
Supplement: Attachment — Submitted filename: reviewers comment_PLOS.docx [file pone.0344242.s003.docx]

5 December. 2025

**Reviewer's comments:**

- In the introduction give examples of the Geographical Indication (GI) certified products from different counries or cultures.
- Under each graph/chart write the full name of the abbreviations, and symbols.
- Please review and clarify the sentence in line 177 and 178 because "relationship between GI product purchasing attitudes and healthy eating attitudes (H1) and between healthy eating attitudes (H2)".
- **2.1.2. Population and Sample**
- In Line 196 "1385 students enrolled in the Nursing Faculty", not clear. Does it mean that the sample was taken from faculty who were previously students in the same university?. or this is the name of the nursing school?
- The explanation of teh sampling seems like stratefied sampling but it needs to be mentioned clearly in the methods.
- In line 224 , you need to mention the type of reliability coefficient. Is that Cronbach alpha or another reliability measure.
- The heading **2.1.4. Data Collection is repeated twice in line 244 and 245**
- **Provide a reference supporting this statement "**Hypotheses were considered supported if the t-value was greater than 1.96 ".
- In Structural Equation Modeling (SEM), **fit indices** are sused as tatistics that assess how well the theoretical model matches the observed data, with key ones including **Chi-Square 𝜒2)**, **RMSEA** (Root Mean Square Error of Approximation), **CFI** (Comparative Fit Index), **TLI** (Tucker-Lewis Index), and **SRMR** (Standardized Root Mean Residual). At least some of them should be measured and added to support the validity of the suggested models.
- In the dada analysis of quantitative data, Using R alone is not sufficient in judging the hypothetical models. There are some
- In qualitative data collection: no mention about reaching saturartion in data collection. It needs to be addressed.
