# Supplementary material for: Geographical indications and health-conscious behaviors among nursing students: A mixed methods study
Source: PLoS One. 2026 Mar 5;21(3):e0344242. doi: 10.1371/journal.pone.0344242 (PMC12962537; doi:10.1371/journal.pone.0344242)
Supplement: Attachment — Submitted filename: 1.Response to Reviewers.docx [file pone.0344242.s004.docx]

**Reviewer's comments:**

*We would like to thank the reviewers for their thoughtful and constructive feedback. Their suggestions were highly relevant and helped improve the overall structure, clarity, and scholarly contribution of the manuscript. All recommended revisions have been implemented accordingly.*

**Reviewer 1**

**Response**

| **In the introduction give examples of the Geographical Indication (GI) certified products from different counries or cultures.** | *Geographical Indication (GI) certified products are becoming an important choice for health-conscious consumers. These products are associated with specific regions, produced using traditional methods, and are perceived as more natural, less processed, and nutritionally superior due to their characteristics (Belletti et al., 2017). For instance, different countries have various GI-certified products: France has Camembert cheese (PDO), Germany is known for Schwarzwalder Schinken, Italy produces Prosciutto di Parma, Spain has Jamón Serrano and Galician Beef, India certifies Vripakshi Hill bananas, Kodaikanal Hill garlic, and Madurai Malli jasmine, Thailand offers Doi Tung coffee and Thai Hom Mali rice, and Turkey protects products such as cherries, pomegranates, grapes, beans, and watermelons (references). Studies show that GI-certified products are considered healthier due to their pure ingredients and traditional production processes (Fernández-Zarza et al., 2021; Török et al., 2020).* |
| --- | --- |

**Response**

| **Under each graph/chart write the full name of the abbreviations, and symbols.** | **Table 1.** Nursing Students' Attitudes Towards HEAS, MES and ASP-GICP Based on Demographic Characteristics  ***Notes:*** *HEAS = Healthy Eating Attitude Scale; MES = Mindful Eating Score; ASP-GICP = Attitude towards Selection of Geographically Indicated Certified Products*  **Table 2.** The Effects of ASP-GICP and MES on Nursing Students' HEAS  ***Notes: ASP-GICP=Attitude towards Selection of Geographically Indicated Certified Products; MES=Mindful Eating Score; HEAS=Healthy Eating Attitude Scale***  **Table 3.** The Role of MES on the Effect of the ASP-GICP on HEAS ***Notes:*** *MES=Mindful Eating Score; ASP-GICP=Attitude towards Selection of Geographically Indicated Certified Products; HEAS=Healthy Eating Attitude Scale* |
| --- | --- |

**Response**

| **Please review and clarify the sentence in line 177 and 178 because "relationship between GI product purchasing attitudes and healthy eating attitudes (H1) and between healthy eating attitudes (H2)".** | *In the first step, the aim was to determine whether there is a significant direct relationship between GI product purchasing attitudes and healthy eating attitudes (H1), and whether healthy eating attitudes significantly influence GI product purchasing behavior (H2)* |
| --- | --- |

**Response**

| **2.1.2. Population and Sample**  **In Line 196 "1385 students enrolled in the Nursing Faculty", not clear. Does it mean that the sample was taken from faculty who were previously students in the same university?. or this is the name of the nursing school?** | *The study population consisted of 1.385 students who were officially registered in the Nursing Faculty of Atatürk University during the 2023–2024 academic year.* |
| --- | --- |

**Response**

| **The explanation of teh sampling seems like stratefied sampling but it needs to be mentioned clearly in the methods.** | *During the sampling process, a list of students enrolled in the 2023–2024 academic year was obtained from the faculty administration. Using a stratified random sampling method, 100 students were randomly selected from each class year, resulting in a total of 400 students invited to participate in the study. The study was completed with 392 students, as 8 participants did not fully complete the survey.* |
| --- | --- |

**Response**

| **In line 224 , you need to mention the type of reliability coefficient. Is that Cronbach alpha or another reliability measure.** | *In this study, the Cronbach's alpha reliability coefficient of the scale was recalculated as 0.86.* |
| --- | --- |

**Response**

| **2.1.4. Data Collection is repeated twice in line 244 and 245** | *Thank you for your valuable comment. However, we respectfully would like to clarify that the heading “2.1.4. Data Collection” appears only once in the revised manuscript. We have carefully rechecked the document, and no repetition of this heading was detected in lines 244–245.* |
| --- | --- |

**Response**

| **Provide a reference supporting this statement "Hypotheses were considered supported if the t-value was greater than 1.96 ".** | *The hypotheses were considered supported when the t-value exceeded 1.96, which corresponds to the critical value for a two-tailed test at the 0.05 significance level (Field, 2018; Hair et al., 2019).*  *Field, A. (2024). Discovering statistics using IBM SPSS statistics. Sage publications limited.*  *Hair, J. F. (2009). Multivariate data analysis.* |
| --- | --- |

**Response**

| **In Structural Equation Modeling (SEM), fit indices are sused as tatistics that assess how well the theoretical model matches the observed data, with key ones including Chi-Square 𝜒2), RMSEA (Root Mean Square Error of Approximation), CFI (Comparative Fit Index), TLI (Tucker-Lewis Index), and SRMR (Standardized Root Mean Residual). At least some of them should be measured and added to support the validity of the suggested models.**  **In the dada analysis of quantitative data, Using R alone is not sufficient in judging the hypothetical models. There are some**  **In qualitative data collection: no mention about reaching saturartion in data collection. It needs to be addressed.** | *The quantitative data collected from the study were analyzed using SPSS (Statistical Package for the Social Sciences) version 22 and AMOS (Analysis of Moment Structures) software. A combination of descriptive statistics and Structural Equation Modeling (SEM) was used to examine the relationships among the variables, such as geographical indication (GI) product purchasing, mindful eating, and healthy eating attitudes. Descriptive statistics were applied to summarize participants’ sociodemographic characteristics (e.g., age, gender, education level, income). Measures of central tendency (mean, median) and variability (standard deviation, range) were calculated for continuous variables, whereas frequency distributions were used for categorical variables. SEM analysis was conducted in two stages. First, the measurement model was evaluated to verify the validity and reliability of the latent constructs. Second, the structural model was tested to assess the hypothesized relationships among variables. Model adequacy was examined using commonly recommended fit indices, including the Chi-square (χ²) statistic, the Comparative Fit Index (CFI), the Tucker–Lewis Index (TLI), the Root Mean Square Error of Approximation (RMSEA), and the Standardized Root Mean Residual (SRMR). These indices were used to determine whether the proposed model demonstrated an acceptable level of fit to the observed data. The hypotheses were considered supported when the t-value exceeded 1.96, which corresponds to the critical value for a two-tailed test at the 0.05 significance level (Field, 2018; Hair et al., 2019). The R² values were calculated for the dependent variables (healthy eating attitudes) to indicate the proportion of variance explained by the independent variables (GI product purchasing, mindful eating, and food awareness). Higher R² values indicate stronger explanatory power of the model. In addition, quantitative model evaluation was not based on R software alone; instead, SEM-based fit indices and parameter estimates were used to ensure a comprehensive assessment of the proposed hypothetical models, as recommended in the SEM literature.* |
| --- | --- |

**Reviewer 2**

**Response**

| **Consider shorter focused title ‘Geographical Indications and Health-Conscious Behaviors Among Nursing Students: A Mixed Methods Study** | *Geographical Indications and Health-Conscious Behaviors Among Nursing Students: A Mixed Methods Study* |
| --- | --- |

**Response**

| **Include specific results e.g., percentages or statistical significance** | *This study examined the relationship between nursing students' awareness of Geographically Indicated (GI) certified products, mindful eating behaviors, and healthy eating attitudes. Using the Theory of Planned Behavior as the framework, it was hypothesized that positive attitudes toward GI products, shaped by social norms and perceived behavioral control, would enhance students' healthy eating behaviors. A cross-sectional study was conducted with 392 nursing students from a faculty population of 1,385. Data were collected using scales measuring healthy eating attitudes, mindful eating, and attitudes toward purchasing GI-certified products. Quantitative findings showed that younger students (17–25 years) and females had significantly higher scores in GI product attitudes and mindful eating (p<0.01). Students with greater knowledge of GI products and those with healthier personal eating habits demonstrated more positive healthy eating attitudes. Structural Equation Modeling revealed that attitudes toward purchasing GI products (S.P.T=0.32, t=3.10, R²=0.10) and mindful eating (S.P.T=0.29, t=2.90, R²=0.08) significantly predicted healthier eating attitudes. Furthermore, mindful eating exhibited a significant moderating effect, strengthening the positive impact of GI product attitudes on healthy eating attitudes (β=0.27, t=3.10, p=0.003). Qualitative interviews highlighted the influence of societal and familial norms on students’ perceptions of GI products, while cost and limited accessibility emerged as key barriers. The study concludes that integrating GI product awareness and mindful eating practices into nursing education may strengthen students’ health-conscious behaviors and support their development as future health professionals.* |
| --- | --- |

**Response**

| **Over all the manuscript needs editing for language.** | *The manuscript has been thoroughly revised for English language, grammar, and clarity throughout the text.* |
| --- | --- |

**Response**

| **The introduction should be more brief and to the point, include recent literature.** | *The Introduction has been revised to be more concise, and recent literature has been included.* |
| --- | --- |

**Response**

| **Consider removing the hypotheses...** | *We appreciate this suggestion; however, the hypotheses were retained to preserve the theoretical coherence of the study and to clearly link the study aims with the analytical framework.* |
| --- | --- |

**Response**

| **The methods section need to be reorganized to reflect one study. The findings should only report the findings and the discussion section needs to be revised. Findings should be discussed within the context of the literature. A brief conclusion would be beneficial. The reference style should be according to journal requirements.** | *The manuscript has been thoroughly revised accordingly: the Methods section was reorganized, Findings and Discussion were clearly separated, findings were discussed in relation to the literature, a brief Conclusion was added, and references were formatted according to journal guidelines.* |
| --- | --- |

**Response**

| **Cite WHO correctly** | *The WHO citation has been corrected and is now cited appropriately as (WHO, 2022).* |
| --- | --- |

**Response**

| **Lines 51–55 repetitive** | *Repetition in lines 51–55 was removed and the text was revised accordingly.*  *Certification and labeling of GI products, including the use of official logos, enhance consumer confidence by signaling quality and authenticity, thereby influencing purchasing decisions and supporting healthier food choices (Calboli, 2015; Vandecandelaere et al., 2021).* |
| --- | --- |

**Response**

| **This section on conceptual framework is not necessary. I suggest you Delete it** | *The conceptual framework section was removed.* |
| --- | --- |

**Response**

| **Are you using conscious eating practices interchangeably with mindful eating ? This is confusing….** | *Terminology has been standardized to “mindful eating” throughout the manuscript..* |
| --- | --- |

**Response**

| **Already used abbreviation before...continue using abbreviation** | *Abbreviation use has been standardized throughout the manuscript.* |
| --- | --- |

**Response**

| **Clarify what HEAS and MES stand for in the figure** | ***Figure 1.*** *Consumer Behavior Toward Geographically Indicated Products Based on the Theory of Planned Behavior. HEAS = Healthy Eating Attitude Scale; MES = Mindful Eating Scale.* |
| --- | --- |

**Response**

| **Either stick to TPB abbreviation or write out theory of planned behavior each time….don’t shift back and forth** | *Usage has been standardized to “TPB” throughout the manuscript.* |
| --- | --- |

**Response**

| **Do not interpret findings in this section** | *Interpretation was removed from this section.* |
| --- | --- |

**Response**

| **Be consistent with the use of tenses, use passive voice and make more direct statement of the findings.** | *Tense usage was standardized to past tense and passive voice.* |
| --- | --- |

**Response**

| **From line 41 to 185 reorganize the literature so it all comes under the Introduction heading. Currently there are a lot of redundancies and there is no need to explain the theory of planned behavior per se. instead use it to organize the literature and then provide a clearer rationale for the study and why the relationship of GI products and health behaviors particularly are important for nursing students.** | *The literature was reorganized under the Introduction heading, redundancies were removed, and TPB was used to structure the literature rather than being explained in detail. The study rationale was clarified accordingly.* |
| --- | --- |

**Response**

| **Move section on how analysis was done to the analysis subsection under Materials and Methods of the manuscript. the analysis of data is typically included in the methodology section, where researchers outline their analytical strategies and justify their choice of methods. When you rewrite this section brake down the analysis steps more explicitly to make it easier for readers to follow.** | *The analysis section was relocated to Materials and Methods and rewritten with clearer* |
| --- | --- |

**Response**

| **Methods: I suggest you combine study 1 and study 2 when you write this section. This is Mixed methods research (Sequential Explanatory Design) and then you can subdivide to 1. Quantitative component and 2. qualitative component instead of study 1 and study 2** | *The Methods section was reorganized as a mixed methods study (Sequential Explanatory Design), with quantitative and qualitative components presented as subsections.* |
| --- | --- |

**Response**

| **Include a brief justification for why you chose a sample size of 400, even though the calculated sample size was 301. And State the response rate** | *During the sampling process, a list of students enrolled in the 2023–2024 academic year was obtained from the faculty administration. Although the minimum required sample size was 301, a larger sample (n=400) was selected to ensure adequate representation of each class year and to account for possible non-response. Using a stratified random sampling method, a total of 400 students 100 from each class year were randomly selected and invited to participate in the study. The study was completed with 392 students, as 8 students did not fully complete the survey. The response rate was 98%.* |
| --- | --- |

**Response**

| **If you calculated the reliability coefficient in your study you should remove the reference from here...I think it should precede this sentence.** | *The reference was repositioned accordingly.* |
| --- | --- |

**Response**

| **Specify how many interviewers collected the data and if they had training...** | *The data were collected by two researchers through face-to-face interviews* |
| --- | --- |

**Response**

| **The use of hypotheses in this qualitative part of the study is not appropriate...please remove them and write emeging themes only.** | *Hypotheses were removed from the qualitative section, and emerging themes are presented instead.* |
| --- | --- |

**Response**

| **Too long paragraph, divide it** | *The paragraph was divided into three paragraphs.*  *Semi-Structured Interview Form*  ***In this study, a semi-structured interview form was developed to deeply understand nursing students' knowledge, attitudes, mindful eating practices, and healthy eating behaviors related to geographical indication (GI) products. The development process began with a comprehensive review of the existing literature on GI products, mindful eating, and healthy eating. In this stage, findings and recommendations from previous studies, academic articles, theses, and relevant books were taken into consideration.***  ***Open-ended questions were prepared to explore participants’ knowledge, attitudes, and behaviors regarding GI products. These questions were designed to allow participants to express their thoughts freely while remaining focused on the main research topics. Flexibility was maintained during question development to encourage sharing of thoughts, feelings, and experiences, while ensuring alignment with the research objectives.***  ***The interview form was piloted with 2–3 participants to assess clarity and the appropriateness of responses. During this phase, the comprehensibility of the questions and the relevance of participants’ answers were evaluated, leading to the refinement of the form by removing unnecessary items and making certain questions more descriptive. To ensure content validity, the draft form was reviewed by expert academics or researchers in the field. Necessary revisions were implemented based on expert feedback. In addition, an internal consistency assessment was conducted to determine whether the questions measured the same construct (Kvale, 2009).*** |
| --- | --- |

**Response**

| **Use participants’ responses consistently instead of participants’ statements** | *Terminology was standardized to “participants’ responses.”* |
| --- | --- |

**Response**

| **Start the discussion by describing your demographic findings...then discuss you main findings from the tools you used and add your qualitative findings appropriately. Always compare and contrast with the literature...** | *The Discussion section was reorganized as suggested, and findings are discussed in comparison with the literature.* |
| --- | --- |

**Response**

| **If this is you finding remove the reference, use references appropriately to build your arguments** | *The reference was removed where the statement reflected the study’s own findings.*  *For instance, participants emphasized that knowledge about the background and stories behind GI-certified products enhances their willingness to purchase them (Almoraie et al., 2024).* |
| --- | --- |

**Response**

| **Reorganize the discussion, add more information about the qualitative findings and discuss within the context of recent literature** | *The Discussion section was reorganized, qualitative findings were expanded, and recent literature was incorporated.* |
| --- | --- |

**Response**

| **Limitations**  **Throughout the manuscript there is shifting between geographical indication (GI) products and GI products. Either spell out consistently or just may GI products throughout after first abbreviation.** | *Terminology was standardized to “GI products” throughout the manuscript after first definition.* |
| --- | --- |

**Response**

| **From here on it is not about implications for research. Put appropriate heading and summarize the following 3 paragraphs. Add a brief conclusion the study.** | *The section was revised by adding an appropriate heading, summarizing the subsequent three paragraphs, and incorporating a concise conclusion to the study.* |
| --- | --- |

**Response**

| **References**  **Format Vancouver style as per journal policy** | *In accordance with the journal's policy, sources are formatted according to Vancouver style.* |
| --- | --- |

*Dear Editor and Dear Reviewers; Thank you very much for your constructive and guiding suggestions.* *Merry christmas.*

*16.01.2026*

*Esin and Erkan.*
